# Short-Term Partial Replacement of Corn and Soybean Meal with High-Fiber or High-Protein Feedstuffs during Metabolizable Energy Assay Influenced Intestinal Histomorphology, Cecal Short-Chain Fatty Acids, and Selected Nutrient Transporters in 21-Day-Old Broiler Chickens

**DOI:** 10.3390/ani12172193

**Published:** 2022-08-26

**Authors:** Oluyinka A. Olukosi, Iyabo W. Oluseyifunmi, Yang Lin, Siara S. Zedonek

**Affiliations:** Department of Poultry Science, University of Georgia, Athens, GA 30602, USA

**Keywords:** metabolizable energy assay, fiber, digestive physiology, broiler chickens

## Abstract

**Simple Summary:**

During metabolizable energy evaluation for feedstuffs, the feedstuffs being tested are usually incorporated into a standard diet. High levels of substitution of the test feedstuff into the standard diet inevitably result in changes to the chemical profile of the newly composited diet. Such changes may result in very high levels of fiber, protein, or other components, but the effects of such changes on growth performance, gut health, or nutrient transport in the intestine are usually not investigated. In the current study, the inclusion of low-protein soybean meal, wheat bran, soy hull, corn gluten feed, or rice bran resulted in increased soluble and insoluble fibers as well as protein content of the final diet depending on feedstuff incorporated into the standard diet. The chemical changes also resulted in, relative to the standard diet, enhanced or depressed weight gain, nutrient utilization, nutrient transporter mRNA, and profile of cecal short-chain fatty acids. Therefore, it is important to consider the gut health influence of test feedstuffs in test diets for metabolizable energy experiments.

**Abstract:**

The current study was conducted to investigate the influence of short-term feeding of test diets during metabolizable energy assays on growth performance, nutrient utilization, jejunal histomorphology, cecal short-chain fatty acids, and nutrient transporters in broilers. One hundred twenty-six broiler chickens were assigned to six treatments, each with seven replicates. Experimental diets were fed between days 14 and 21. Treatments included a corn–soybean meal reference diet and five test diets with low-protein soybean meal (LPSBM), wheat bran, soy hull, corn gluten feed, or rice bran. Birds were weighed on days 14 and 21; excreta, cecal content, and jejunal tissues were collected on day 21. Seven-day weight gain was highest (*p* < 0.01) for birds receiving the reference diet or LPSBM, whereas FCR was lowest (*p* < 0.05) for birds receiving the soy hull diet. Cecal acetate and total short-chain fatty acids were higher (*p* < 0.05) for wheat bran compared with the soy hull test diet. Jejunal villi were longer (*p* < 0.05) for chickens receiving the reference diet or LPSBM test diet. Glucose transporter (GLUT1) mRNA was greater (*p* < 0.05) in broilers receiving rice bran compared with soy hull test diets. Therefore, when reporting energy assays, it is important that indicators of animal growth or gut health be included to help contextualize energy utilization.

## 1. Introduction

An assay for metabolizable energy of feedstuffs using the difference method necessitates the replacement of a reference diet with a test feedstuff [1,2]. Any practical (i.e., not purified) diet formulated to meet the nutrient requirement of the birds can be used as a reference diet [3,4,5,6], and practical diets are generally preferred to semi-purified ones [2,3]. The reference diet is then substituted with a sizeable quantity of the test feedstuff. The level of test feedstuff to be incorporated into the reference diets is determined both by how much of the feedstuff can be practically incorporated and other practical considerations such as palatability.

Many metabolizable energy assays use at least 300 g/kg inclusion of test feedstuff in the reference diet [4,7,8,9]. Adeola and Ileleji [3] used up to 600 g/kg inclusion of maize-DDGS and, as the authors explained, such high inclusion levels may be necessary to enable a reliable determination of the ME value of test feedstuff. Larbier and Lecleerq [10] indicated that the standard error of the simultaneous linear equations used for the “difference” method is greater when lower inclusion levels of assay feedstuffs are used and that AME determination became sufficiently accurate when an inclusion level of, at least, 300 g/kg is used. Olukosi [6] showed that this proposition is feedstuff-dependent, and lower inclusion levels of certain feedstuffs (with inclusion limited by palatability, rather than handling, e.g., oil, issues) have been used in literature with apparent success [11,12].

Nevertheless, high levels of substitution of practical reference diets with test feedstuffs usually precipitate drastic changes in the chemical profile of the test diet and often result in depressed growth performance. Information on growth performance is not usually included in metabolizable energy assays, but as Adeola and Ileleji [3] pointed out, such depression in growth performance may be an inevitable consequence of diet dilution. Clearly, the extent of growth depression will depend on the chemical profile of the test feedstuff in relation to the reference diet and on the changes in the final chemical composition of the final diet.

Consequent modification of the chemical composition of the final diet as a result of substituting the reference diet with test feedstuff sometimes results in flooding of the intestinal tract with high levels of crude protein, fat, fiber, or other substances. It can be argued that this represents what would normally take place if animals were to consume the feedstuff solely. Nevertheless, it is important to investigate how the substitution of a reference diet with high levels of test feedstuff might influence growth performance, and attempt to account for possible physiologic changes that account for the depression in performance. The objective of the current experiment was to study the impact of partial substitution of corn–soybean meal reference diet with test feedstuffs of varying chemical compositions on short-term growth performance, jejunal histomorphology, cecal microbial products, and expression of selected gene markers for nutrient transport and intestinal integrity.

## 2. Materials and Methods

Procedures used in the experiment reported here were approved by the Institutional Animal Care and Use Committee of the University of Georgia.

**Animals, experimental design, and diets.**

A total of one hundred twenty-six male broilers (off-sex Cobb 500) were used in this experiment. On day 0, birds were assigned to six treatments; each treatment had seven replicates with three birds per replicate. All the birds received a corn–soybean meal broiler starter diet formulated to meet Cobb 500 nutrient requirements (3011 kcal/kg AME and 217 g/kg crude protein) from day 0 until 14 days of age. Birds were allocated into cages according to a randomized complete block design to ensure that the average body weight for each treatment was similar on day 0 (44.3 g).

The six experimental diets provided from day 14 to day 21 were a corn–soybean meal (SBM) reference diet and five test diets in which low-protein SBM (LPSBM), wheat bran, soy hull, corn gluten feed, or rice bran, at the rates of 400, 600, 500, 500, or 500 g/kg, respectively, was added to a basal diet. The LPSBM was produced by mixing SBM (480 g/kg crude protein) with soy hull at the rate of 10.1:1 with anticipated final CP content in LPSBM of 440 g/kg. In order to make the experimental test diets, a basal diet consisting of corn, SBM, and soybean oil (energy-yielding feedstuffs) was first mixed. The reference and five test diets were subsequently made by mixing the required quantities of the other micro-ingredients, as well as the test feedstuffs, with the basal diet. This was done to ensure that all the diets had similar levels of Ca, non-phytate P, and limiting digestible amino acids. Titanium dioxide (5 g/kg) was added to all the diets as an indigestible marker. The chemical and NIR-analyzed compositions of the test feedstuffs are shown in Table 1. The feedstuffs and calculated chemical compositions of the basal, reference, and test diets are shown in Table 2, whereas the analyzed composition of the reference and test diets are shown in Table 3.

The broiler chickens were placed in 42 battery cages in an environmentally controlled room. The temperature (93 °C on day 0 and decreased step-wise to 78 °C on day 19) and lighting regime (24 h of light on day 0, decreased step-wise to 16 h of light and 8 h of dark on day 19) used were in accordance with Cobb 500 recommendation. A room-length automatic ventilation system was used in the experiment room. Feed and water were provided ad libitum. Pre-experimental diets were fed from day 0 to day 14 whereas experimental diets were fed from day 14 to day 21.

### 2.1. Growth Performance Measurement

Birds and feed provided were weighed on days 0, 14, and 21, and leftover feed was weighed on days 14 and 21. Weight gain and feed intake were corrected for mortality before calculating feed conversion ratio (FCR) for the experimental (day 14 to day 21) phase.

### 2.2. Samples Collection

All the birds were euthanized at the end of the experiment at 21 days of age. For jejunal histomorphology, a 2-cm section was incised from the mid-jejunum of two birds per pen and fixed in formalin pending further processing. For measurement of gene expression of selected genes, jejunal tissues collected from two birds per pen were snap-frozen in liquid nitrogen for further processing. Cecal content from both ceca of 2 birds per cage was collected and stored at −20 °C before analysis for short-chain fatty acids (SCFAs).

### 2.3. Chemical Analysis

Diets and feedstuffs samples were oven-dried and then ground (0.5 mm) for analysis of dry matter (DM), nitrogen (N), gross energy (GE), and neutral and acid detergent fibers. All analyses (except otherwise stated) were performed according to AOAC [13] methods. Samples for dry matter determination were dried in a drying oven at 100 °C for 24 h (Method 934.01). The combustion method was used for nitrogen analysis (Method 968.06). Ankom 200 Fiber Analyzer (Ankom Technology, Macedon, NY, USA) was used for acid detergent fiber (ADF) and neutral detergent fiber (NDF) analysis. Gross energy analysis was performed using an isoperibol bomb calorimeter with benzoic acid (calibration standard). The processing of the jejunal tissue for histomorphological examination followed procedures previously described by Greiner et al. [14]. Analysis of cecal SCFAs was performed using gas chromatography (GC) according to the method described previously [15]. Briefly, 3 mL of deionized water was used to dilute 1 g of cecal content. The resulting solution was centrifuged at 10,000× *g* for 10 min, and the supernatant was removed and mixed with metaphosphoric acid (25%). Ethyl acetate was then added to the mixture (1:2), vortexed, and allowed to settle. The supernatant in the vortexed mixture was analyzed for SCFAs using GC after being transferred to a glass vial.

### 2.4. Quantitative Real-Time PCR Assay

Gene expression of proteins for tight junctions and intestinal nutrient transporters was analyzed using QT-PCR. Homogenization of jejunal tissue was performed using QIAzol lysis reagent (QIAGEN, Hilden, Germany) followed by extraction of total RNA. The RNA was converted to cDNA after quantitation and dilution. Real-time PCR reaction was performed with reaction master mix iTaq Universal SYBR Green Supermix (Bio-Rad, CA, USA) in a Step One Plus real-time PCR system (Thermo Fisher Scientific, MA, USA). The data (in duplicates) were analyzed using the 2^(−ΔΔCt)^ method [16]. All the primers, including housekeeping and target genes, are listed in Table 4.

**Statistical analysis.**

The data were analyzed by the mixed model procedure of SAS (SAS Institute Inc., Cary, NC, USA) as appropriate for a randomized complete block design. The blocks (replicates) were treated as random variables whereas the treatments were fixed variables in the model. Growth performance data were collected between days 14 and 21 (the experimental period). Although the birds had similar body weight on day 0 and received the same diet until day 14, there was a significant difference in body weight at the start of the experimental phase on day 14. Therefore, day 14 body weight was used as a covariate in the analysis of growth performance data. Significantly different means for all responses were separated using Tukey. Significance was declared when *p* ≤ 0.05.

## 3. Results

The analyzed composition of test feedstuffs showed that soy hull had the lowest level of essential amino acids, except for lysine which was lowest for corn gluten feed (Table 1). Analysis with NIR showed that soy hull had the highest quantity of acid and neutral detergent fibers, total dietary fiber, and total and insoluble non-starch polysaccharides. On the other hand, wheat bran had the highest NIR-analyzed total arabinoxylan, whereas rice bran had the highest NIR-analyzed lignin level.

Weight gain over the 7-day experimental period was highest (*p* < 0.01) for the birds receiving corn–SBM reference diet and the LPSBM test diet (Table 5). Weight gain was lowest, whereas feed intake was highest (*p* < 0.01), for birds receiving the soy hull test diet, thus resulting in the highest FCR (*p* < 0.01) for that treatment. Feed intake was similar between birds receiving LPSBM and soy hull test diets. Dry matter retention was lower (*p* < 0.05) for birds receiving the soy hull test diet compared with those receiving the reference diet or LPSBM test diet. Nitrogen retention was higher (*p* < 0.01) for birds receiving the wheat bran test diet compared with all other diets except the rice bran test diet. Diet AME and AMEn were higher (*p* < 0.01) for the LPSBM test diet compared with all other treatments except for the reference diet and rice bran test diet (AMEn). Generally, soy hull and corn gluten feed test diets had significantly (*p* < 0.01), or tendency for (*p* < 0.10), lowest AME and AMEn.

There were no treatment effects on the cecal content of propionate, whereas isobutyrate tended to be lower (*p* = 0.073) in birds receiving the wheat bran test diet (Table 6). Cecal acetate and total SCFAs were higher (*p* < 0.05) in birds receiving the wheat bran test diet compared with birds receiving soy hull, corn gluten feed, or rice bran test diets. Except for the valerate content, birds receiving corn gluten feed test diet had lower (*p* < 0.01), or tended to have lower (*p* < 0.10), SCFAs than other diets. In addition, cecal butyrate content was higher (*p* < 0.05) for birds receiving the LPSBM test diet compared with soy hull or corn gluten feed test diets.

Jejunal villi were longer (*p* < 0.05) in broiler chickens receiving the corn–soybean meal reference diet and LPSBM test diet compared with broiler chickens receiving the wheat bran or soy hull test diets (Table 7). Crypts were deeper (*p* < 0.01) in broiler chickens receiving LSPBM compared with those receiving soy hull test diets. There were no treatment effects on villi width or villi height:crypt depth.

Among the genes tested, treatment effects were only observed for the basolateral-side glucose transporter (GLUT1), which was higher in expression (*p* < 0.05) in broilers receiving rice bran than in those receiving soy hull test diets (Table 8). There were no treatment effects observed for gene expression of the epithelial tight junction gene (CLDN1), apical-side glucose transporter (SGLT1), or cationic amino acid transporter (CAT2).

## 4. Discussion

An assay for metabolizable energy for feedstuffs involves feeding of the test feedstuff solely (direct method) or partial replacement of a “reference” diet with the test feedstuff (to produce assay or test diet) when “indirect” assay methods are employed [2]. Almost invariably, the chemical composition of the test feedstuff is different from the reference diet, leading to different chemical profiles for the reference and test diets. It is reasonable to predicate the determination of metabolizable energy of test feedstuffs on differences in chemical profiles of reference and test diets in as much as these differences are expected to result in energy utilization differences. The part that is often unaccounted for is the impact that these differences in chemical profiles may have on the intestinal milieu and how this may help explain effects seen in nutrient and energy utilization. Therefore, the objective of the current study was to examine the influence of partial substitution of a corn–SBM reference diet with test feedstuffs of varying chemical compositions (high fiber or protein) on short-term growth performance, jejunal histomorphology, cecal microbial products, and expression of selected genes as markers for nutrient transport and intestinal integrity. The diets were formulated to have similar levels of macro- and microminerals and vitamins in order to forestall possible nutritional imbalances that usually result from dilution of a reference diet with test feedstuffs during AME assays [2].

Substitution of the corn–SBM reference diet with wheat bran or soy hull resulted in markedly increased (>5-fold) ADF, or NDF being increased up to 3-fold, in respective test diets. On the other hand, the substitution of the reference diet with LPSBM, corn gluten feed, or rice bran had very little effect on the fiber contents of the respective test diets, but the LPSBM test diet dramatically increased protein content relative to the reference diet. These chemical profile differences were largely responsible for differences in weight gain of the birds, which was most pronounced for the test diet with 500 g/kg soy hull, which also had some of the lowest levels of metabolizable energy and retention of dry matter and N. It is noteworthy that the test diet with soy hull also had the largest feed intake, which was arguably due to the increased bulkiness of the soy hull test diet. The growth performance data were collected over a period of seven days, and thus the observation of dramatic weight gain effects of the test diets within a relatively short time indicates that the effects of the test diets were not inconsequential. Therefore, possible explanations for these growth performance effects are warranted.

Chemical analysis of the diets revealed that although gross energy was only marginally different among the diets, as is usually the case, there were large differences in the profiles of crude protein and fiber components. These differences will influence nutrient intake and hence the quantity of, for example, crude protein and fiber passing to the proximal and distal parts of the digestive tract. In the current experiment, further calculations showed that N intake was 80 g in the LPSBM test diet compared with 49 g in the reference diet. On the other hand, N digestibility was six percentage points lower in LPSBM compared with the reference diet. Depending on the quantity of carbohydrates and protein reaching the hindgut, the dynamics of fermentation may shift toward favoring proteolytic or fibrolytic microorganisms. Such a dynamic shift will influence the quantity, and profile, of SCFAs and branched-chain fatty acids [17,18,19,20]. In the current experiment, isovalerate was the only branched-chain fatty acid (BCFA) that was influenced by dietary treatments, with quantity of the BCFA being lowest in test diets with wheat bran. Branched-chain fatty acids, in contrast to the SCFAs, are produced from microbial fermentation of nitrogenous compounds [21,22]. In the current experiment, the protein:total fiber (ADF + NDF) was 0.37 for the wheat bran test diet, the second lowest in the experiment. Therefore, it can be expected that the substantially higher proportion of fiber relative to protein in the hindgut digesta was greater for birds receiving the wheat bran test diet compared with the other diets. This would suggest, therefore, that the lower isovalerate (indicative of protein fermentation) content in those birds receiving the wheat bran test diet was driven by comparatively lower protein, relative to fiber, in the cecal content of the birds receiving the diet, as has also been observed in a human model [23].

In the current experiment, soy hull and LPSBM test diets had the highest content of isovalerate, a BCFA which is indicative of protein fermentation. Further calculations showed that these two diets had the lowest NDF:ADF ratio, compared to the other diets. The implication of this fiber ratio is the relative proportion of soluble to insoluble fiber components reaching the hindgut. The more soluble fibers are more rapidly fermented because of their greater hydration capacity and hence could contribute more to SCFA production during fermentation [24,25]. In an in vitro fermentation model, fermentation of wheat bran proceeded faster, and to a larger extent, than fermentation of soy hull [26] leading to the conclusion that fiber level and type influence microbial fermentation dynamics.

Butyrate, on the other hand, is generally considered a beneficial SCFA because of its involvement in colonocyte metabolic activities [17,27,28]. In the current experiment, the test diets containing soy hull and corn gluten feed generally had lower butyrate in the cecal content. Cecal butyrate content is largely driven by fiber fermentation [29,30] and hence may be impeded by either: (1) decreased quantitative availability of fermentable fiber [17,29], exemplified by the lower NDF:ADF ratio of the soy hull test diet in the current study, or (2) comparatively higher protein:total fiber as in the corn gluten feed test diet in the current experiment [17]. The implications of these are that both quantitative and qualitative assessments of protein, fiber, and fermentable carbohydrates in test diets during metabolizable energy assays are important in interpreting the data inasmuch as these differences may confer differences in the metabolizable energy value of feedstuffs [25,31].

Villi length and crypt depth characteristics have been used by many authors as indications of optimum gut health [32,33]. In the current experiment, there were no treatment effects on villi height:crypt depth, and therefore the villi height and crypt depth responses must be interpreted in that context. Compared with the control diet, the villi were shorter in birds receiving wheat bran or soy hull test diets. At the same time, crypts were shallowest in birds receiving the soy hull test diet. These two diets were the ones with the highest content of NDF and ADF, and it seems, therefore, that these components influenced the development of the absorptive ability of the small intestine.

The impact of fiber on intestinal morphology is widely reported [34,35,36]. Soluble fibers that promote increased digesta viscosity are generally known to stunt the growth of villi and increase cellular turnover leading to disproportionately deep crypts [36,37]. In the current experiment, even though villi were shorter in birds receiving wheat bran and soy hull test diets, the crypt depths were not disproportionally different from those of the other birds. The implication of the effect seen on villi height, even in the absence of the crypt depth effect, will translate to growth performance metrics due to the connection between villi and nutrient intake. As is often the case, the observation in the current experiment is that, to a large extent, the pattern of villi height response to dietary treatments was reflected in the weight gain response to the treatments.

Investigation of treatment effects on nutrient transporters in the current experiment was limited to four genes, two of which are known to mediate the transport of glucose and one of which is responsible for the transport of cationic amino acids. The treatment effects observed in the current experiment were limited to GLUT1, a glucose transporter that is largely expressed in the basolateral side of the intestinal epithelium. The expression pattern indicates lower mRNA abundance of the transporter in birds receiving the soy hull test diet (birds receiving this test diet also had some of the lowest AME and AMEn values). Although the expression of SGLT1 (apical-side glucose transporter) was not significantly influenced by the treatments, the mRNA abundance was similarly lowest in birds receiving the soy hull test diet. Expression patterns of glucose transporters are sometimes associated with dietary fiber content [38,39,40] which may subsequently affect energy availability [39]. Rather than, or in addition to, a direct response to dietary fiber content, it is likely that nutrient transporter expression patterns are in response to other stimuli (e.g., intestinal hormones or other luminal factors) that are modulated by dietary fibers [41,42,43]. Therefore, it can be reasoned that differences in the chemical profile of feedstuffs influence the pattern of nutrient transporter expression with a consequence on the growth of the birds as well as nutrient and energy utilization.

## 5. Conclusions

It was concluded from the current experiment that the substitution of test feedstuffs in a reference diet during a metabolizable energy assay has a substantial effect on the growth performance, cecal microbial fermentation products, and digestive physiology of broiler chickens. Therefore, when reporting energy assays, it is important to include some indicators of animal growth performance, digestive physiology, or gut health as they may help put energy utilization in context. This is especially relevant when different assays utilize different inclusion levels of, for example, the same test feedstuff.

## Figures and Tables

**Table 1:** Analyzed composition (g/kg) of the test feedstuffs.

Items	LPSBM	Wheat Bran	Soy Hull	Corn Gluten Feed	Rice Bran
Dry matter	900	902	914	897	902
Crude protein	434	138	89.2	158	125
Analyzed essential amino acids					
Arg	36.1	12.7	4.68	7.76	12.8
His	13.6	5.11	2.67	6.07	4.45
Ile	25.8	5.90	4.46	7.20	5.82
Leu	39.7	10.6	7.02	16.2	10.6
Lys	33.4	7.72	7.13	6.41	7.87
Met	7.08	2.50	1.11	3.15	3.31
Phe	27.0	7.04	3.90	7.65	6.73
Thr	19.9	5.56	3.79	7.65	5.70
Trp	6.50	2.04	0.56	0.90	1.14
Val	25.8	8.51	4.79	9.78	8.44
Near-infrared reflectance (NIR) analyzed composition					
Starch	-	144	88.1	465	229
NDF	-	426	457	243	249
ADF	-	123	274	107	117
Lignin	-	31.2	20.2	18.6	44.6
Total dietary fiber	-	381	641	211	143
Total NSPs	-	346	578	157	119
Insoluble NSPs	-	296	492	133	102
Total AX	-	222	121	84.7	60.6

NDF—neutral detergent fiber; ADF—acid detergent fiber; NSPs—non-starch polysaccharides; AX—arabinoxylan.

**Table 2:** Feedstuff and nutritional composition (g/kg as fed) of experimental diets.

Items	Basal Diet	Reference Diet	LPSBM	Wheat Bran	Soy Hull	CGF	Rice Bran
Basal diet		951.8	549.9	345.2	443.9	450.8	448.5
Corn	670.0						
Soybean meal	280						
Soybean oil	50						
Di-calcium phosphate		18.5	16.5	14.5	19.5	10.0	14.0
Limestone		6.6	6.6	9.3	1.0	3.0	1.0
Titanium dioxide		5.0	5.0	5.0	5.0	5.0	5.0
Low-protein SBM (LPSBM)			400.0				
Wheat bran				600.0			
Soy hull					500.0		
Corn gluten feed (CGF)						500.0	
Rice bran							500.0
Vitamin premix ^1^		5.0	5.0	5.0	5.0	5.0	5.0
Trace minerals premix ^2^		5.0	5.0	5.0	5.0	5.0	5.0
Methionine		1.6	2.0	2.5	3.3	3.7	4.5
Lysine		2.0	4.5	6.7	8.5	8.5	8.5
Threonine		0.5	1.5	2.8	4.8	5.0	4.5
Salt NaCl		4.0	4.0	4.0	4.0	4.0	4.0
Total	1000	1000	1000	1000	1000	1000	1000
Calculated composition							
Protein	191	196	291	165	152	137	160
Ca	0.9	8.4	8.4	8.4	8.4	8.4	8.5
P	3.6	6.4	7.1	10.6	5.5	5.7	12.5
Available P	1.2	4.2	4.2	4.2	4.2	4.2	4.2
Na	0.2	1.7	1.7	1.7	1.9	1.8	1.7
Cl	0.3	3.0	2.8	2.6	2.8	2.8	3.1
Digestible amino acids, g/kg							
Ile	8.1	8.1	5.7	5.9	3.8	3.8	3.8
Leu	17.1	14.4	12.0	10.0	6.7	6.8	6.8
Lys	10.1	11.5	11.2	11.2	11.3	11.3	11.3
Met	3.1	4.5	4.3	4.6	4.6	5.1	5.9
Thr	7.2	7.5	7.5	7.9	8.1	8.3	7.8
Trp	2.2	2.6	1.7	1.8	1.2	1.2	1.2
Val	9.1	9.0	6.7	7.0	4.2	4.3	4.2

^1^ Vitamin A, 5484 IU; vitamin D3, 2643 ICU; vitamin E, 11 IU; menadione sodium bisulfite, 4.38 mg; riboflavin, 5.49 mg, d-pantothenic acid, 11 mg; niacin, 44.1 mg, choline chloride, 771 mg; vitamin B12, 13.2 µg; biotin, 55.2 µg; thiamine mononitrate, 2.2 mg; folic acid, 990 µg; pyridoxine hydrochloride, 3.3 mg. ^2^ Iodine, 1.11 mg; manganese, 66.06 mg; copper, 4.44 mg; iron, 44.1 mg; zinc, 44.1 mg; selenium, 300 µg.

**Table 3:** Analyzed composition (g/kg, as fed basis) of the experimental diets.

Diets	Dry Matter	Gross Energy, kcal/kg	Crude Protein	Neutral Detergent Fiber	Acid Detergent Fiber
Reference diet	906	4184	174	112	51
LPSBM test diet	902	4189	265	103	77
Wheat bran test diet	913	4051	159	318	109
Soy hull test diet	910	3930	139	344	257
Corn gluten feed test diet	889	3918	189	195	43
Rice bran test diet	899	4220	158	136	70

**Table 4:** GenBank accession numbers and sequences of forward and reverse primers used for real-time PCR.

Gene Symbol	Accession Number	Full Name	Function	Forward Primer	Reverse Primer
18S	XR_003078042.1	18S ribosomal RNA	Housekeeping gene	AGCCTGCGGCTTAATTTGAC	CAACTAAGAACGGCCATGCA
Beta-actin	NM_205518.1	β-actin	Housekeeping gene	CAACACAGTGCTGTCTGGTGGTA	ATCGTACTCCTGCTTGCTGATCC
CLDN1	NM_001013611.2	Claudin-1	Tight junction	TGGAGGATGACCAGGTGAAGA	CGAGCCACTCTGTTGCCATA
GLUT1 (SLC2A1)	NM_205209.1	Glucose transporter-1	Glucose transporter	CTTTGTCAACCGCTTTGG	CAGAATACAGGCCGATGAT
SGLT1 (SLC5A1)	NM_001293240.1	Sodium glucose transporter-1	Glucose transporter	GCCGTGGCCAGGGCTTA	CAATAACCTGATCTGTGCACCAGT
CAT2 (SLC7A2)	XR_005859133.1	Cationic amino acid transporter-2	Cationic amino acid transporter	TGCTCGCGTTCCCAAGA	GGCCCACAGTTCACCAACAG

**Table 5:** Growth performance (day 14 to day 21), coefficients of total tract retention, and dietary metabolizable energy (kcal/kg) for broiler chickens in response to short-term feeding of high levels of high-protein or high-fiber feedstuffs in a corn–soybean meal reference diet during metabolizable energy assay.

Diets	Gain, g	FI, g	FCR	cDMR	cNR	AME	AMEn
Reference diet	410 ^a^	592 ^ab^	1.45 ^b^	0.68 ^a^	0.54 ^bc^	2772 ^b^	2653 ^ab^
LPSBM (400 g/kg)	480 ^a^	629 ^a^	1.31 ^b^	0.66 ^a^	0.48 ^c^	2916 ^a^	2725 ^a^
Wheat bran (600 g/kg)	246 ^b^	582 ^ab^	2.55 ^b^	0.62 ^ab^	0.60 ^a^	2677 ^bc^	2581 ^b^
Soy hull (500 g/kg)	96.7 ^c^	715 ^a^	7.83 ^a^	0.54 ^b^	0.52 ^bc^	2510 ^d^	2414 ^c^
Corn gluten feed (500 g/kg)	205 ^b^	472 ^bc^	2.32 ^b^	0.61 ^ab^	0.49 ^bc^	2581 ^cd^	2438 ^c^
Rice bran (600 g/kg)	184 ^b^	435 ^c^	2.40 ^b^	0.64 ^ab^	0.56 ^ab^	2725 ^b^	2629 ^ab^
Pooled SEM	20.3	31.6	0.365	0.033	0.016	29.2	30.4
*p* values	<0.001	<0.001	<0.001	0.032	<0.001	<0.001	<0.001

LPSBM—low-protein soybean meal; *n* = 7 cages with 3 birds per replicate cage; ^a–d^ means within a column but with different superscripts are significantly different (*p* < 0.05).

**Table 6:** Cecal short-chain fatty acid (SCFA, mM) composition in response to short-term feeding of high levels of high-protein or high-fiber feedstuffs in a corn–soybean meal reference diet during metabolizable energy assay.

Diets	Acetate	Propionate	Isobutyrate	Butyrate	Isovalerate	Valerate	Total SCFAs
Reference diet	13.9 ^abc^	0.656	0.084	3.40 ^ab^	0.075 ^ab^	0.156 ^ab^	18.3 ^abc^
LPSBM (400 g/kg)	15.6 ^ab^	0.935	0.071	3.74 ^a^	0.081 ^a^	0.179 ^ab^	20.6 ^ab^
Wheat bran (600 g/kg)	17.5 ^a^	0.653	0.024	3.12 ^abc^	0.016 ^ab^	0.113 ^b^	21.4 ^a^
Soy hull (500 g/kg)	12.7 ^bc^	0.601	0.054	1.81 ^c^	0.082 ^a^	0.145 ^ab^	15.4 ^bc^
Corn gluten feed (500 g/kg)	9.99 ^c^	0.689	0.082	1.89 ^bc^	0.074 ^ab^	0.199 ^a^	12.9 ^c^
Rice bran (600 g/kg)	10.7 ^c^	0.621	0.031	3.10 ^abc^	0.032 ^bc^	0.177 ^ab^	14.7 ^bc^
Pooled SEM	1.05	0.1000	0.0174	0.354	0.0166	0.0169	1.37
*p* values	<0.001	0.219	0.073	0.002	0.027	0.020	<0.001

LPSBM—low-protein soybean meal; *n* = 7 cages with 2 birds per replicate cage; ^a–c^ means within a column but with different superscripts are significantly different (*p* < 0.05).

**Table 7:** Jejunal histomorphology (µm) in 21-day-old broilers in response to short-term feeding of high levels of high-protein or high-fiber feedstuffs in a corn–soybean meal reference diet during metabolizable energy assay.

Diets	Villi Height (VH)	Villi Width	Crypt Depth (CD)	VH:CD
Reference diet	1104 ^a^	273	176 ^ab^	6.54
LPSBM (400 g/kg)	1118 ^a^	262	189 ^a^	6.06
Wheat bran (600 g/kg)	826 ^b^	275	144 ^ab^	6.07
Soy hull (500 g/kg)	772 ^b^	263	122 ^b^	6.38
Corn gluten feed (500 g/kg)	958 ^ab^	216	148 ^ab^	6.67
Rice bran (600 g/kg)	953 ^ab^	251	175 ^ab^	5.69
Pooled SEM	56.1	17.7	12.8	0.489
*p* values	<0.001	0.221	0.007	0.738

LPSBM—low-protein soybean meal; *n* = 7 cages with 2 birds per replicate cage; ^a,b^ means within a column but with different superscripts are significantly different (*p* < 0.05).

**Table 8:** Gene expression in the jejunum of 21-day-old broilers in response to short-term feeding of high levels of high-protein or high-fiber feedstuffs in a corn–soybean meal reference diet during metabolizable energy assay.

Diets	CLDN1	GLUT1	SGLT1	CAT2
Reference diet	1.00	1.00 ^ab^	1.00	1.00
LPSBM (400 g/kg)	0.91	1.58 ^ab^	0.78	1.41
Wheat bran (600 g/kg)	0.83	1.23 ^ab^	0.97	1.07
Soy hull (500 g/kg)	0.78	0.98 ^b^	0.62	1.74
Corn gluten feed (500 g/kg)	1.12	1.10 ^ab^	0.87	1.39
Rice bran (600 g/kg)	0.78	1.80 ^a^	0.85	1.47
Pooled SEM	0.123	0.184	0.120	0.287
*p*-value	0.211	0.022	0.460	0.618

LPSBM—low-protein soybean meal; *n* = 7 cages with 2 birds per replicate cage; ^a,b^ means within a column but with different superscripts are significantly different (*p* < 0.05); CLDN1—claudin 1, epithelial tight junction gene; GLUT1—basolateral-side glucose transporter; SGLT1—apical-side glucose transporter; CAT2—cationic amino acid transporter.

## Data Availability

The data presented in this study are available in the supplementary material.

## Supplementary Materials

The following supporting information can be downloaded at: https://www.mdpi.com/article/10.3390/ani12172193/s1.
